# Astragalin Suppresses Inflammatory Responses and Bone Destruction in Mice With Collagen-Induced Arthritis and in Human Fibroblast-Like Synoviocytes

**DOI:** 10.3389/fphar.2019.00094

**Published:** 2019-02-12

**Authors:** Qingyun Jia, Tengteng Wang, Xiaoyun Wang, Hao Xu, Yang Liu, Yongjun Wang, Qi Shi, Qianqian Liang

**Affiliations:** ^1^Longhua Hospital, Shanghai University of Traditional Chinese Medicine, Shanghai, China; ^2^Institute of Spine, Shanghai University of Traditional Chinese Medicine, Shanghai, China; ^3^Key Laboratory of Theory and Therapy of Muscles and Bones, Ministry of Education, Shanghai University of Traditional Chinese Medicine, Shanghai, China; ^4^School of Rehabilitation Science, Shanghai University of Traditional Chinese Medicine, Shanghai, China

**Keywords:** astragalin, rheumatoid arthritis, matrix metalloproteinase, CIA, fibroblast-like synoviocytes

## Abstract

Astragalin, as a bioactive flavonoid with anti-inflammatory, antioxidant, and protective properties, provides a potential agent for rheumatoid arthritis (RA). In this study, its therapeutic efficacy and the underlying mechanisms were explored using DBA/1J mice with collagen-induced arthritis (CIA). It was demonstrated that astragalin could significantly attenuate inflammation of CIA mice. The effects were associated with decreased severity of arthritis (based on the arthritis index), joint swelling and reduced bone erosion and destruction. Furthermore, astragalin treatment suppressed the production of pro-inflammatory cytokines (TNF-α, IL-1β, IL-6, and IL-8), and inhibited the expression of matrix metalloproteinases (MMP-1, MMP-3, and MMP-13) in chondrocytes and synovial cells of CIA mice. Fibroblast-like synoviocytes derived from RA patients (MH7A cells) were applied to verify these effects. *In vitro*, astragalin inhibited the expression of matrix metalloproteinases (MMP-1, MMP-3, and MMP-13) dose-dependently in TNF-α-induced MH7A cells, with no apparent cytotoxicity. Furthermore, astragalin suppressed the phosphorylation of p38, JNK, and the activation of c-Jun/AP-1 in TNF-α-induced MH7A cells. In conclusion, it has proven that astragalin could attenuate synovial inflammation and joint destruction in RA at least partially by restraining the phosphorylation of MAPKs and the activating of c-Jun/AP-1. Therefore, astragalin can be a potential therapeutic agent for RA.

## Introduction

Rheumatoid arthritis (RA) is a chronic autoimmune joint disease, which is characterized by inflammation of synovial tissue and the destruction of bone and cartilage in multiple peripheral joints (Smolen et al., 2016). RA is estimated to affect approximately 1% of the adult population worldwide and is a major source of disability (Myasoedova et al., 2010). Despite traditional disease-modifying anti-rheumatic drugs and biological agents have proven to improved clinical outcomes in patients with RA. However, these beneficial effects are far from satisfaction because of their obvious adverse effects with a high frequency and high cost of treatment (Guo et al., 2018). Therefore, further efforts are required to develop new therapeutic agents with fewer side effects for treatment of RA. Of the potential cellular participants in RA, fibroblast-like synoviocytes (FLSs) play a critical role by regulating the secretion of inflammatory mediators and expression of MMPs, which cause changes in chondrocyte metabolism and matrix degradation (Bartok and Firestein, 2010; Korb-Pap et al., 2016). Hence, exploring new anti-rheumatic arthritis drugs to alleviate the destructive behavior of RA-FLSs may be a reasonable and effective method for the treatment of RA.

Astragalin, also known as kaempferol-3-O-glucoside, is a newly found flavonoid from leaves of persimmon and green tea seeds and has been used for treating many diseases for a long time (Riaz and Rasul, 2018). Several biological functions of astragalin have been studied, including anti-inflammatory, anti-oxidant, and anti-cancer. The previous study has proven that astragalin can downregulate lipopolysaccharide (LPS)-induced inflammatory responses by suppressing the NF-κB signaling pathway (Kim and Kim, 2011; Nhiem et al., 2011). Astragalin can also increase the survival from lethal endotoxemia and reduce acute lung injury in a murine asthma model (Li et al., 2013; Liu et al., 2015). In addition, astragalin can inhibit ovalbumin (OVA)-induced allergic inflammation and eosinophilia in lung tissues (Cho et al., 2014; Kim et al., 2017). However, the therapeutic benefits of astragalin in RA remain unknown. Thus, we speculated that astragalin may be a potential anti-rheumatic arthritis drug.

In this study, we investigated the role of astragalin in the regulation of synovial inflammation and joint destruction in a collagen-induced arthritis (CIA) mouse model *in vivo* and its underlying mechanisms in MH7A RA-derived FLSs *in vitro*.

## Materials and Methods

### Drugs and Reagents

**FIGURE 1:**
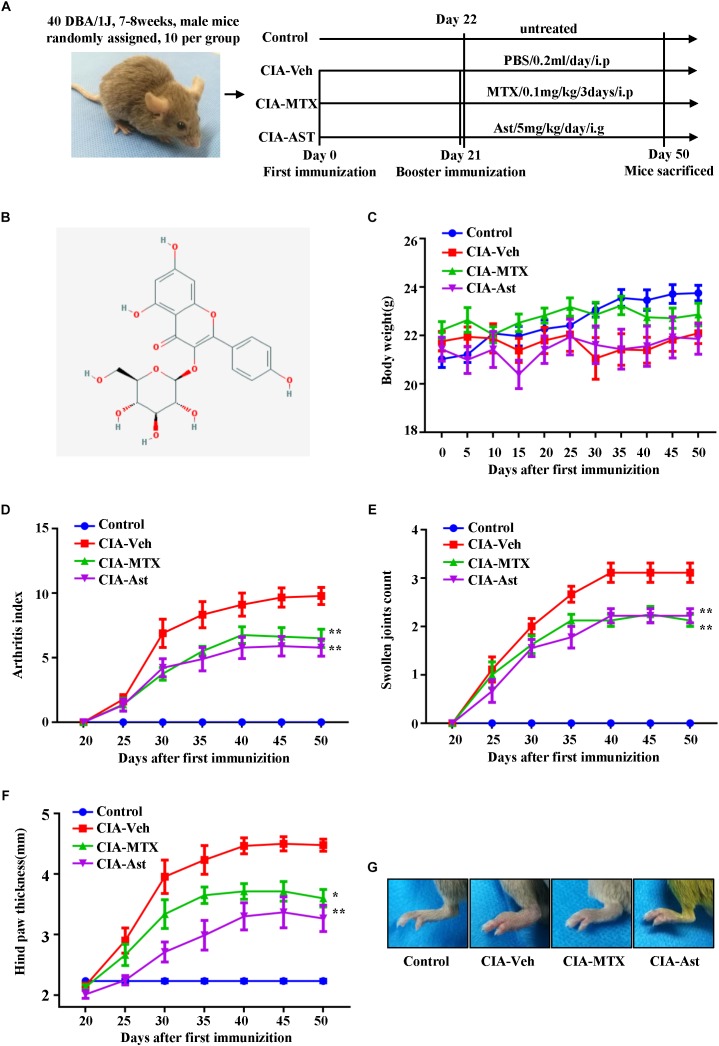
Astragalin attenuated the symptoms of CIA mice. The time diagram of the process of CIA induction and treatment **(A)**. The chemical structure of astragalin **(B)**. Mice with established CIA were injected either with PBS or 0.1 mg/kg/3 day MTX intraperitoneally or with oral dose of astragalin 5 mg/kg/day after day 21 booster immunization. The body weight **(C)**, arthritis index **(D)**, swollen joints count **(E)**, and hind paw thickness **(F)** were scored and recorded every 5 days in a blinded manner. The representative pictures of the hind paws of CIA mice **(G)**. Results are shown as mean ± SEM of ten mice per group. ^∗^*p* < 0.05, ^∗∗^*p* < 0.01 versus CIA-Veh group.

Astragalin (PubChem CID:5282102; purity >98.0%; Figure 1B) was purchased from Chengdu Pufei De Biotech Co., Ltd. (Chengdu, China); methotrexate (MTX) from Shanghai Oriental Medicine Science and Technology Industry Co., Ltd. (Shanghai, China, cat. # 100138); bovine type II collagen (cat. # 20021), Freund’s complete adjuvant (CFA, cat. # 7001), Freund’s incomplete adjuvant (IFA, cat. # 7002) from Chondrex (Redmond, WA, United States); MMP-1 (cat. # ab215083), MMP-3 (cat. # ab189572), MMP-13 (cat. # ab100605) antibodies and ELISA kit from Abcam (Cambridge, United Kingdom); TNF-α (cat. # EMC102a), IL-1β (cat. # EMC001b), IL-6 (cat. # EMC004), IL-8 (cat. # EMC104) ELISA kit from Neobioscience (Shanghai, China); recombinant human tumor necrosis factor (TNF-α) from PeproTech (Rocky Hill, CT, United States, cat. # 300-1A); anti-c-Jun (cat. # 9165), anti-phospho-c-Jun (cat. # 3270), anti-c-Fos (cat. # 2250), anti-phospho-c-Fos (cat. # 5348), anti-p38 (cat. # 8690), anti-phospho-p38 (cat. # 4511), anti-extracellular signal regulated kinase (ERK, cat. # 4695), anti-phospho-ERK (cat. # 4370), anti-c-Jun N-terminal kinase (JNK, cat. # 4252), anti-phospho-JNK (cat. # 4255) from Cell Signaling Technology (Beverly, NJ, United States); goat anti-rabbit IgG H&L (Alexa Fluor^®^ 488) (Abcam, Cambridge, United Kingdom, cat. # ab150077); Actinomycin D (cat. # A1410), 4′,6-diamidino-2-phenylindole (cat. #D9564) from Sigma (Sigma-Aldrich, United States); cell culture medium, fetal bovine serum and trypsin from Gibco (Gland Island, NE, United States).

### Animals

Specific pathogen-free, DBA/1J male mice (7∼8-week-old) were provided by the Vital River company (Beijing, China). Ten of these mice were assigned to the negative control group and thirty to the experimental group. This study was approved by the Medical Ethics Committee of Shanghai University of Traditional Chinese Medicine. The methods applied in this study were carried out in accordance with the approved guidelines and regulations.

### Induction of Collagen-Induced Arthritis

Collagen-induced arthritis model was established according to a previous protocol (Brand et al., 2007). Briefly, bovine collagen type II was dissolved in 10 mM acetic acid to 2 mg/ml. This solution was then emulsified in equal volumes of complete Freund’s adjuvant (CFA, 4 mg/ml M. tuberculosis). CIA mice were immunized intradermally by 100 μl of emulsion at the base of the tail on day 0. To ensure a high incidence of RA induction in the CIA model, 100 μl of bovine type II collagen emulsified in incomplete Freund’s adjuvant was used as a booster on day 21 after the first immunization. Typically, the first signs of arthritis appeared in this model at 21–28 days after the first immunization.

### *In vivo* Drug Administration

DBA/1J mice were randomly divided into four groups (10 mice/group). Group 1 was used the non-immunized mice (Control), whereas mice in group 2–4 were used the CIA mice. Group 2: mice treated with PBS, 0.2 ml/day/intraperitoneally (CIA-Veh); Group 3: mice treated with MTX, 0.1 mg/kg/3 day/intraperitoneally (CIA-MTX); Group 4: mice treated with astragalin, 5 mg/kg/day/intragastrically (CIA-Ast). All the mice from these groups received additional treatments between day 22 and day 50. The time diagram of the process of CIA induction and treatment is shown in Figure 1A.

### Arthritis Assessment

Collagen-induced arthritis was considered to have successfully developed when swelling was observed in at least one digit or paw. The global assessment, arthritis index, swollen joints count, and hind paw thickness were scored and recorded every 5 days in a blinded manner as reported before. The severity of arthritis in each of the four paws was scored with a 0–4 scale by visual evaluation of each paw as follows: 0, no evidence of erythema and swelling; 1, erythema and mild swelling confined to the tarsals or ankle joint; 2, erythema and mild swelling extending from the ankle to the tarsals; 3, erythema and moderate swelling extending from ankle to metatarsal joints; 4, erythema and severe swelling encompass the ankle, foot, and digits, or ankylosis of the limb. The final score for each mouse was the sum of the four paws. Thickness of the ankle was measured with digital calipers placed across the ankle joint at the widest point.

### Ultrasound Analysis

After 7 weeks of treatment, the knee and ankle joints of these mice were examined using the Vevo 2100 imaging system (Vevo LAB, FUJIFILM Visual-Sonics, Toronto, ON, Canada). Both B mode and color Doppler were scanned with the 550 scan head, 40–50 MHz probes, wall filter = 3 mm/s, scan speed = 2 mm/s, dynamic range = 65.0 dB, the pulses to radiofrequency cycle number = 2, the pulse repetition frequency = 6 kHz, after the 2-dimensional (2D) images were obtained in real time, the images were analyzed and measurements manually determined and calculated using the Vevo LAB software studio measurement package. The Vevo LAB software was then used to construct the scans into a 3-dimensional (3D) image, which allowed for accurate volume measurement and image sculpting creating a visual representation of the knee and ankle joints.

### Histopathological Assessment

On day 50, mice were sacrificed, the left knee and ankle joint tissues were collected and fixed in 4% paraformaldehyde, then decalcified in 10% EDTA for 20 days. Thereafter, the tissues were embedded in paraffin and sectioned using routine methods, and stained with hematoxylin and eosin (H&E). The joint sections were measured using a scale of 0–3 for grading the synovial inflammation, pannus formation, and destruction of bone and cartilage.

### Micro-Computed Tomography (Micro-CT) Imaging

On day 50, the mice were sacrificed, and their right legs were excised and fixed in 4% formalin for 1 day. A micro-CT (SCANCO μCT80) was used to estimate the structural status in the right knee and ankle joints. The X-ray tube voltage was 55 kV and, the current was 72 μA, with a voxel size of 10 μm. The segmented images were 3D-reconstructed using the SCANCO proprietary software. Joint bone radiological destruction was scored on a scale from 0 to 3: 0, no damage; 1, minor; 2, moderate; 3, severe.

### Immuno-Histochemical Analysis

The above-described paraffin-embedded knee and ankle joint tissues were deparaffinized with ethanol and xylene. After being hydrated in PBS, tissues were blocked with 0.3% hydrogen peroxide (H_2_O_2_) for 10 min. Next, the tissues were incubated with primary antibodies against MMP-1, MMP-3, MMP-13 at 4°C overnight. After washing the tissues with PBS for three times, secondary antibodies were added and incubated for another 1 h; then the signal was amplified with HRP-conjugated streptavidin using a Vectastain Elite ABC kit (Vector, United States).

### Cell Culture

MH7A, a human RA-FLSs cell line (Miyazawa et al., 1998), purchased from the Riken cell bank (Tsukuba, Japan), was gifted by the Chinese Academy of Sciences, Shenzhen. The cells were maintained in 1640 medium (Hyclone, United States) with 100 IU/ml of penicillin/streptomycin (Sigma-Aldrich, United States) and 10% heat-inactivated fetal bovine serum (Gibco, United States) in an incubator with 5% CO2 at 37°C.

### Cell Viability Assay

Cell viability was determined using the CCK-8 assay as described previously (Jia et al., 2015). Briefly, MH7A cells were seeded at 5^∗^10^3^ cells/well in 96-well culture plates, incubated overnight, and then exposed at various concentrations of astragalin for 24, 48, and 72 h. Then, CCK-8 (Dojindo, Japan) was added to each well of the plate and incubated at 37°C for 1.5 h. The resulting optical density was detected at 450 nm by a microplate reader.

### Quantitative RT-PCR Analysis

MH7A cells were pretreated with astragalin (0, 50, 100, 200 μM) for 2 h and then incubated for another 24 h in the presence or absence of TNF-α (10 ng/mL). Total RNA was extracted using Trizol reagent according to the manufacturer’s instructions and each sample was reverse transcribed using the cDNA synthesis kit according to the manufacturer’s protocol. Real-time PCR analysis was performed using SYBR Green PCR Premix Ex Taq II reagents on a CFX96 real-time system (Thermo Fisher Scientific, United States). Relative gene expression was calculated by the ^ΔΔ^Ct method. The primer sequences (forward and reverse) were as follows: for MMP-1, 5′-CTCAATTTCACTTCTGTTTTCTG-3′ and 5′-CATCTCTGTCGGCAAATTCGT-3′; for MMP-3, 5′-GGCTTCAGTACCTTCCCAGG-3′ and 5′-GCAGCAACCAGGAATAGGTT-3′; for MMP-13, 5′-CAAGATGCGGGGTTCCTGAT-3′, 5′-AATGCCATCGTGAAGTCTGGT-3′.

### Enzyme Linked Immunosorbent Assay

On day 50, the mice were sacrificed, serum samples were extracted from peripheral blood and stored at -80°C until analysis. MH7A cells were seeded into 6-well plates (1^∗^10^6^ cells/well) for 24 h, then pretreated with astragalin (0, 50, 100, 200 μM) for 2 h, then incubated for another 24 h in the presence or absence of TNF-α (10 ng/ml). Cell culture supernatants were collected and stored at -80°C until analysis. The concentrations of the cytokines in serum and culture supernatants were determined by ELISA using a commercial kit according to the manufacturer’s instructions.

### Western Blot Analysis

MH7A cells were pretreated for 2 h with various concentrations of astragalin (0, 50, 100, 200 μM), and then exposure to TNF-α (10 ng/ml) for 0.5 h. The protein was collected, 30 μg of protein from each sample was separated by 15% SDS-PAGE and transferred to polyvinylidene fluoride membrane. After blocking with 5% bovine serum albumin in TBST at room temperature for 2 h, the membranes were incubated with the corresponding primary antibodies overnight at 4°C. After washing with TBST for three times, the membranes were incubated with the secondary antibodies. Proteins were scanned using the ECL detection system. The gray values of protein bands were analyzed using ImageJ software.

#### Immunofluorescence Analysis

MH7A cells were seeded on a round coverslips in 24-well plates for 24 h, and then pretreated with astragalin (200 μM) for 2 h, stimulated with TNF-α for 0.5 h, fixed with 4% paraformaldehyde for 15 min and then permeated with 0.2% Triton X for 20 min. After blocking with 5% bovine serum albumin at room temperature for 0.5 h, cells were incubated with the anti-phospho-c-Jun antibody overnight at 4°C and then incubated with the goat anti-rabbit IgG H&L secondary antibody for 1 h at room temperature. The nuclei were visualized using 4′,6-diamidino-2-phenylindole (DAP). The coverslips were mounted onto glass slides and images were recorded by an Olympus BX-51 microscope.

### Statistical Analysis

The data were reported as mean ± standard error of the mean (SEM). Significant differences were assessed using GraphPad Prism 7.0 software (GraphPad, La Jolla, CA, United States). One-way ANOVA followed by Dunnett’s *t*-test were used to determine differences between groups. As to three groups treated at different times points, two-way ANOVA comparison was performed. *p* < 0.05 was considered statistically significant.

## Results

### Astragalin Attenuated the Symptoms of CIA Mice

To investigate the therapeutic effects of astragalin on RA *in vivo*, we established CIA mouse model. Treating mice with astragalin initiated at 22 days after the first immunization. Assessment of the severity of the arthritis was observed every 5 days after the booster injection. Compared with the negative control group, the CIA model mice showed a slight body weight loss (Figure 1C), and with higher scores in arthritis index, the number of swollen joints, and hind paw thickness. Both astragalin and MTX treatment had positive effects on attenuating the arthritis index, swollen joints count, and hind paw thickness of CIA mice (Figure 1D–G). These results indicated that astragalin effectively inhibited the development of arthritis.

### Astragalin Inhibited Joint Space Widening and Synovial Vascularity

**FIGURE 2:**
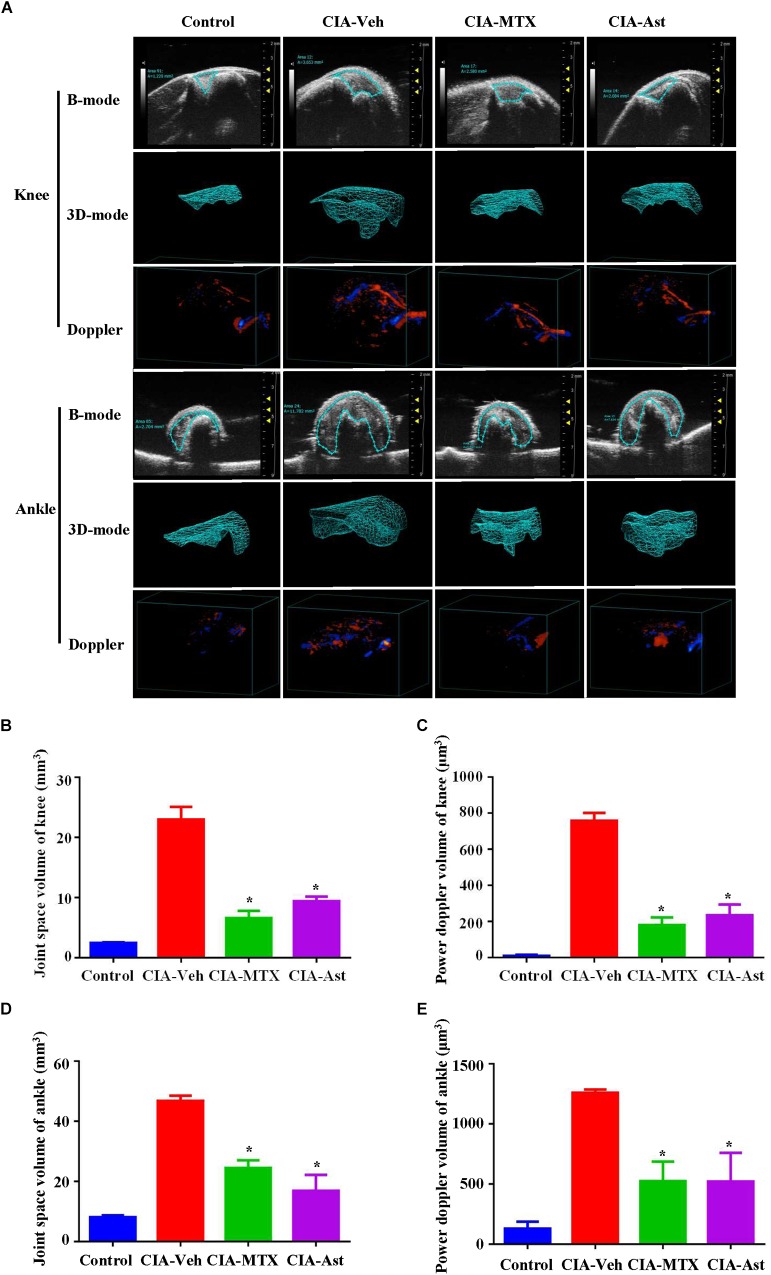
Astragalin inhibited joint space widening and synovial vascularity. After 7 weeks of treatment, the knee joints and the ankle joints **(A)** were analyzed using the Vevo 2100 imaging system in both B mode and color doppler mode. 3D reconstructions of knee and ankle joints using the Vevo LAB software, which allowed for accurate volume measurement of the joint space widening and synovial vascularity **(B–E)**. Data are shown as mean ± SEM of four mice per group, ^∗^*p* < 0.05, ^∗∗^*p* < 0.01 versus CIA-Veh group.

Ultrasound (US) in combination with the Doppler technique has already been used to quantify synovial inflammation by measuring the increased joint space volume and blood flow in CIA mice (Clavel et al., 2008; Elhai et al., 2015). The volumetric changes of joint space and vascularity in knee and ankle were measured with the Vevo 2100 imaging system. After 7 weeks of treatment, the knee and ankle joints of these mice were evaluated. The results of 3D high-frequency ultrasonography and color Doppler indicated that astragalin significantly inhibited joint space widening and synovial vascularity in the knee and ankle joints (Figure 2A–E).

### Astragalin Alleviated Synovial Inflammation and Joint Destruction

**FIGURE 3:**
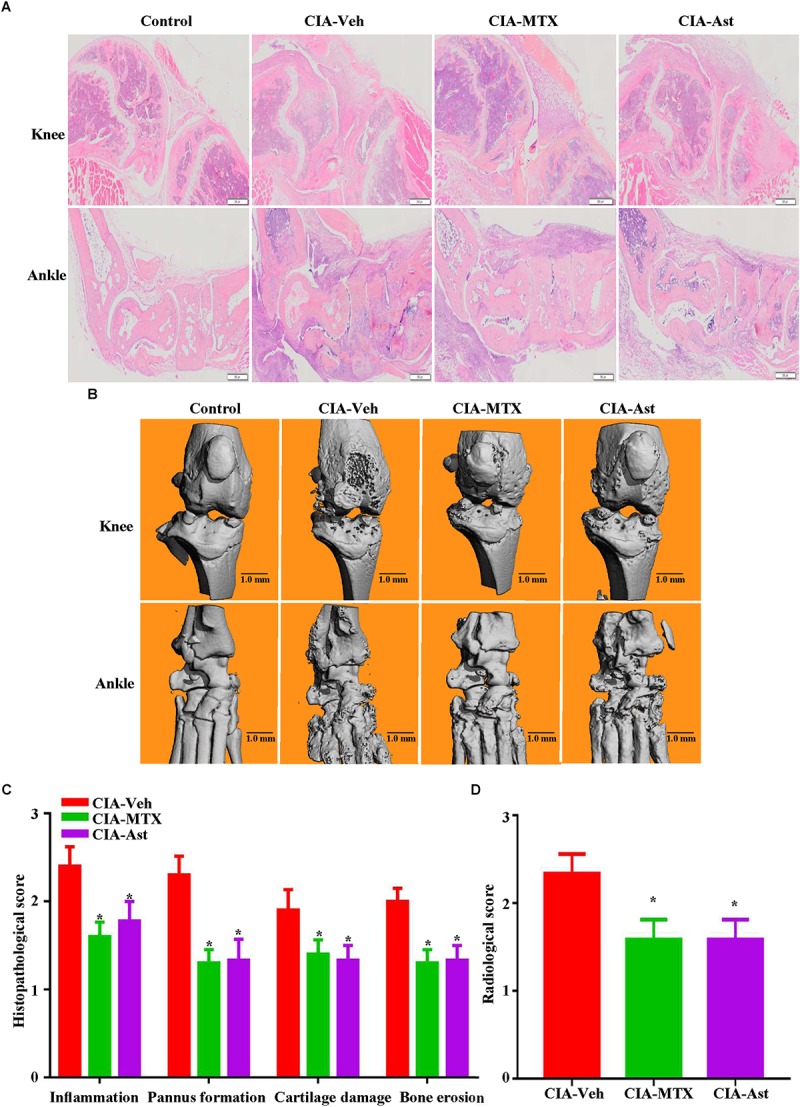
Astragalin alleviated synovial inflammation and joint destruction. On day 50, mice were sacrificed, the knee and ankle joints stained with hematoxylin and eosin **(A)**. 3D reconstructions of the micro-CT analysis from CIA mice using the SCANCO proprietary software **(B)**. The histopathological severity was assessed and calculated **(C)**. Bone radiological destruction scores were attributed from the micro-CT analysis **(D)**. Data are shown as mean ± SEM of ten mice per group, ^∗^*p* < 0.05 versus CIA-Veh group.

A histopathological evaluation of the knee and ankle joints was performed to examine the degrees of arthritic damage. Severe synovial hyperplasia, infiltration of inflammatory cells into synovial tissues, pannus formation, and cartilage and bone destruction were observed in the CIA mice. As expected, MTX significantly attenuated the pathological symptom of CIA in the knee and ankle joints. Consistently, astragalin also alleviated the histopathological arthritic damage in the CIA joints (Figure 3A,B). The 3D reconstruction of a micro-CT analysis of knee and ankle joints showed astragalin treatment markedly diminished bone destruction compared with untreated CIA control mice (Figure 3C,D).

### Astragalin Reduced the Production of Pro-inflammatory Cytokines and the Expression of MMPs in Inflamed Joints of CIA Mice

**FIGURE 4:**
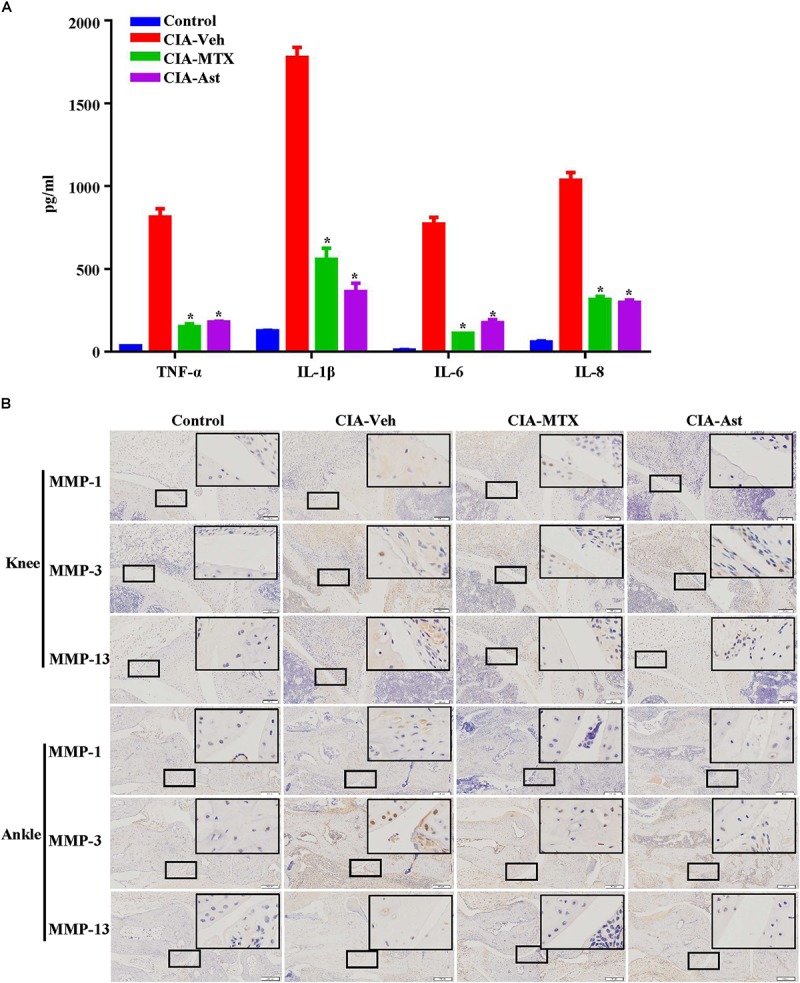
Astragalin reduced the production of pro-inflammatory cytokines and the expression of MMPs in inflamed joints of CIA mice. The serum level of TNF-α, IL-1β, IL-6, and IL-8 were determined using the ELISA assay **(A)**. Expression of MMP-1, MMP-3 (100×) and MMP-13 at the sites of cartilages and synovial tissues in the knee joints and the ankle joints **(B)** were measured by immune-histological staining. Representative images are shown from three independent experiments. Data are shown as mean ± SEM of ten mice per group, ^∗^*p* < 0.05, ^∗∗^*p* < 0.01 versus CIA-Veh group.

Various cytokines regulate the pathogenesis of RA. The production of pro-inflammatory cytokines, particularly TNF-α, IL-1β, IL-6, and IL-8, may cause synovial inflammation and pain. MMP-1, MMP-3, and MMP-13 are essential factors for the degradation of joint cartilages. In the present experiment, the production of pro-inflammatory cytokines in the serum of CIA mice was determined using the ELISA kit, and the expression of MMPs in knee and ankle joint tissues were measured by immune-histochemical staining. As shown in Figure 4A, the serum levels of TNF-α, IL-1β, IL-6, and IL-8 were significantly lower in the astragalin-treated group than those in CIA-Veh group. The expression levels of MMP-1, MMP-3, and MMP-13 in cartilages and synovial tissues notably increased in CIA mice and reduced after the treatment of astragalin and MTX (Figure 4B).

### Astragalin Suppressed the mRNA and Protein Expression of MMPs in TNF-α-Induced MH7A Cells

**FIGURE 5:**
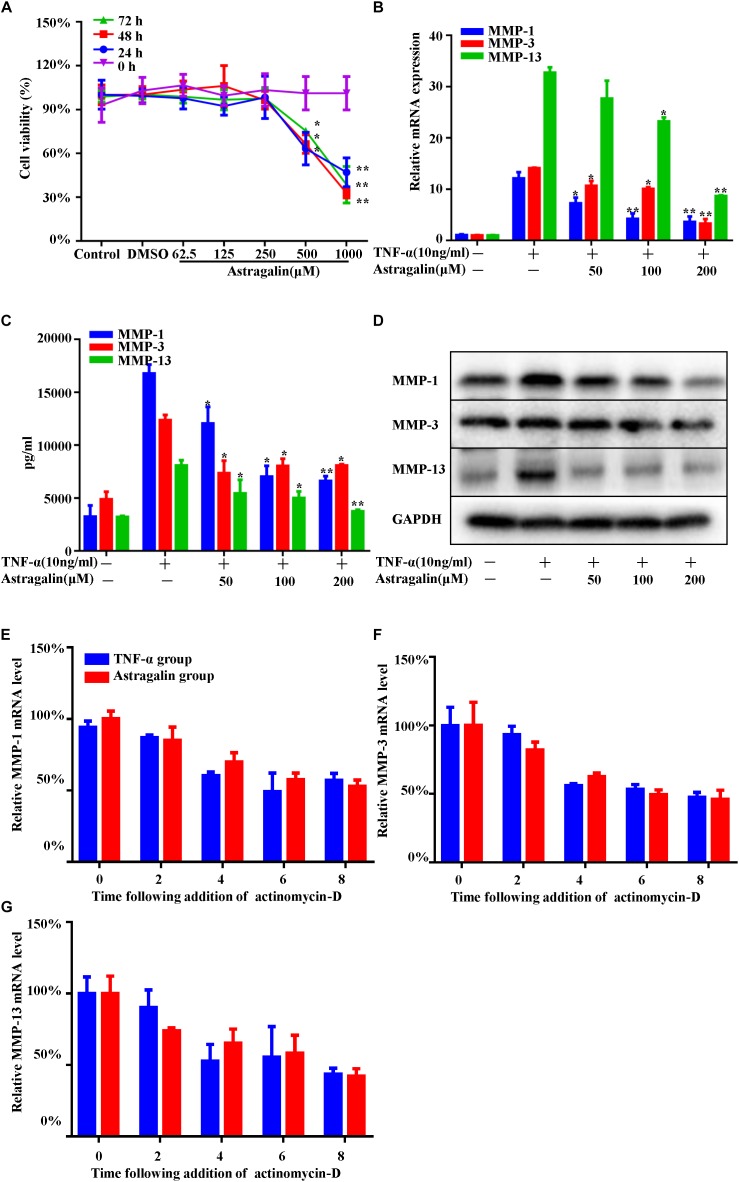
Astragalin suppressed the mRNA and protein expression of MMPs in TNF-α-induced MH7A cells. MH7A cells were treated with different concentrations of astragalin for 0, 24, 48, and 72 h, and cell viability was measured by CCK-8 assay **(A)**. MH7A cells pretreated for 2 h with various concentrations of astragalin (0, 50, 100, 200 μM), and then exposure to TNF-α (10 ng/ml) for 24 h, the mRNA and protein levels of MMP-1, MMP-3, and MMP-13 were determined using the RT-PCR, ELISA assay, and western blot analysis **(B–D)**. MH7A cells were pretreated with astragalin (200 μM) or not for 2 h, then stimulated with TNF-α for 6 h. Actinomycin D (4 μg/mL) was added to the cells and then mRNA was isolated at time point 0, 2, 4, 6, and 8 h. The levels of MMPs mRNA stability were detected by RT-PCR **(E–G)**. Data are shown as mean ± SEM of three independent experiments. ^∗^*p* < 0.05, ^∗∗^*p* < 0.01 compared with TNF-α stimulation alone.

MMPs are mainly secreted by FLSs, which play a critical role in the destruction of joint cartilage (Burrage et al., 2006; Araki and Mimura, 2017; Malemud, 2017). Stimulating FLSs with TNF-α or IL-1 *in vitro* increases MMPs production. To assess the potential cytotoxicity of astragalin, cell viability was evaluated by the CCK-8 assay. As shown in Figure 5A, astragalin did not affect cell viability, even at a concentration as high as 250 μM after 72 h incubation. Therefore, we decided to set the highest concentration of astragalin at the 200 μM for the following experiments. To determine the protective effect of astragalin on MMPs expression, MH7A cells were incubated for 24 h with TNF-α. As shown in Figure 5B–D, TNF-α significantly increased MMPs expression both in mRNA and protein levels. Astragalin significantly and dose-dependently decreased the mRNA and protein expression of MMP-1, MMP-3, and MMP-13 in TNF-α-induced MH7A cells compared to the group treated with TNF-α alone.

In addition, the expression of MMPs is affected by transcriptional regulation and mRNA stability. To clarify the effect of astragalin on the mRNA stability of MMPs, we performed mRNA stability assays. MH7A cells were pretreated with astragalin (200 μM) or not for 2 h, then stimulated with TNF-α for 6 h. Actinomycin D (4 μg/mL) was added to the cells and then mRNA was isolated at time point 0, 2, 4, 6, and 8 h. The levels of MMPs mRNA stability were detected by RT-PCR. We found that astragalin did not alter the mRNA stability of MMPs (Figure 5E–G).

### Astragalin Inhibited TNFα-Induced Activation of MAPK and AP-1 Pathways

**FIGURE 6:**
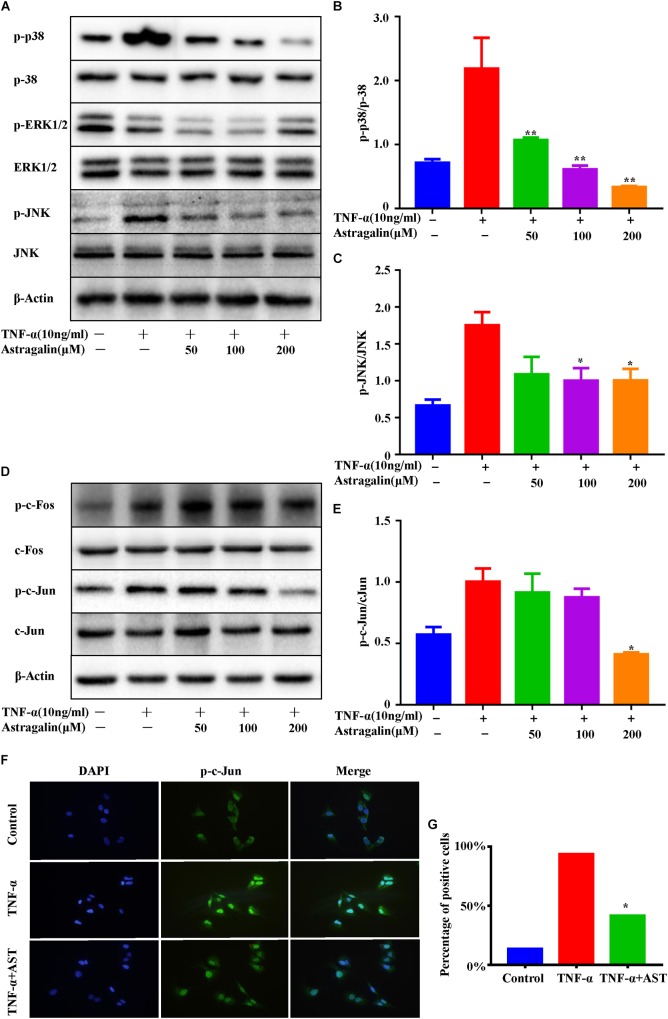
Astragalin inhibited TNFα-induced activation of MAPK and AP-1 pathways. MH7A cells pretreated for 2 h with various concentrations of astragalin (0, 50, 100, 200 μM), and then exposure to TNF-α (10 ng/ml) for 0.5 h, the total and phosphorylated levels of JNK, ERK1/2, and p38 were determined by Western blot analysis **(A–C)**. The total and phosphorylated levels of c-Jun, c-Fos were analyzed by Western blot analysis **(D,E)**. MH7A cells pretreated with astragalin (200 μM) or not for 2 h, and then exposure to TNF-α (10 ng/ml) for 0.5 h. Immunofluorescence analysis was used to detect intracellular localization of p-c-Jun. Representative 400× images **(F)**. More than 200 cells from each sample were measured **(G)**. Data are shown as mean ± SEM of three independent experiments. ^∗^*p* < 0.05, ^∗∗^*p* < 0.01 compared with TNF-α stimulation alone.

Previous studies have shown that the MAPK and AP-1 mediated pathways can regulate the expression of MMPs synthesis and inflammatory cytokines (Schett et al., 2000). To further investigate the mechanisms of astragalin inhibiting production of MMPs, we examined the effect of astragalin on the MAPK and AP-1 mediated pathways in TNF-α-induced MH7A cells. MH7A cells were pretreated with various concentrations of astragalin for 2 h, stimulated with TNF-α (10 ng/ml) for 30 min. Then, the levels of the phosphorylation of MAPK (ERK, JNK, and p38), and the levels of c-Fos and c-Jun (a component of AP-1) were detected using western blot. Astragalin treatment significantly inhibited the phosphorylation of JNK and p38, but not ERK, in a dose-dependent manner (Figure 6A–C). The phosphorylation of c-Jun was decreased in TNF-α-induced cells by astragalin in a dose-dependent manner. However, it did not affect the phosphorylation of c-Fos (Figure 6D,E). In addition, the immunofluorescence analysis revealed that astragalin reduced the TNF-α-induced p-c-Jun protein expression and its translocation to the nucleus (Figure 6F,G).

## Discussion

Astragalin is an extract separated from leaves of persimmon and green tea seeds, which possess potent biological effects including anti-inflammatory, antioxidant and anticarcinogenic activities (Riaz and Rasul, 2018). It shows ability to inhibit the production of inflammatory mediators, such as IL-1β, IL-6, and TNF-α in macrophages and in mice with allergic asthma (Nhiem et al., 2011; Li et al., 2013; Liu et al., 2015). These studies suggested benefits of astragalin for the treatment of inflammatory disorders. However, to our knowledge, no study has investigated its anti-rheumatic arthritis effect on animal models or humans. In the present study, we demonstrated for the first time that astragalin effectively inhibited the worsening of synovial inflammation and joint destruction in mice with CIA. These beneficial effects may occur via inhibiting the destructive behaviors of rheumatoid FLSs. RA is a chronic inflammatory joint disease, which is characterized by synovial hyperplasia, infiltration of inflammatory cells into synovial tissue as well as the subsequent erosion of cartilage and bone (Smolen et al., 2016). CIA model is the most commonly used autoimmune model of RA (Brand et al., 2007). The main pathological features of CIA include synovium hyperplasia with infiltration of inflammatory cells, pannus formation, cartilage degradation, and erosion of bone. In this study, we found that astragalin markedly attenuated arthritis symptom in CIA mice. At the dose of 5 mg/kg/day, a strong anti-arthritic property of astragalin was manifested by a decrease in CIA-induced paw edema and swelling as observed in arthritis score. US has proven to be a valuable method for evaluating arthritic lesions in DBA/1J mice (Clavel et al., 2008). In the present study, ultrasound and color Doppler analysis suggested that the increase of joint space volume and vascularity in the knee and ankle joints were significantly attenuated by astragalin treatment. The results of the histological examination and micro-CT scan of the knee and ankle joints further confirmed that synovial hyperplasia, pannus formation, cartilage damage, and bone erosion were significantly attenuated by astragalin.

Destruction in articular cartilage and bone hallmarks the presence of RA (Myasoedova et al., 2010). Osteoclasts are major effectors of the destruction of cartilage and bone, but recent studies suggested that the destruction is primarily caused by FLSs (Huber et al., 2006; Bartok and Firestein, 2010). FLSs from the intimal lining are major inducers of cartilage destruction in RA for that they can produce enormous degradative enzymes. Among the various classes of proteinases produced by the inflamed synovium, MMPs are particularly significant (Boissier, 2011; Araki and Mimura, 2017). MMPs are a large group of enzymes that play critical roles in the destruction of articular cartilage and bone due to their ability to degrade a wide variety of extracellular matrix components (Burrage et al., 2006; Aikawa et al., 2008). Collagenases (MMP-1, MMP-13) and stromelysins (MMP-3) are especially important in RA (Green et al., 2003; Iwamoto et al., 2008; Araki et al., 2016). Their synthesis and activation are motivated by various factors, such as pro-inflammatory cytokines, growth factors, matrix proteins, and reactive oxygen species. Activated RA-FLSs are the major source of MMPs and inflammatory mediators in the synovial tissue. Therefore, the inhibition of MMPs can significantly reduce RA-FLSs cartilage invasiveness. Regulation of MMPs gene expression and activation is a good parameter for assessing disease activity in humans and evaluates the effects of various agents on inflammatory conditions, both *in vitro* and *in vivo* experiments. In the present study, we found that CIA mice treated with astragalin had lower histopathology scores, inflammation, synovial hyperplasia, articular cartilage, and bone destruction. At the same time, astragalin significantly down-regulated the expression levels of MMP-1, MMP-3, and MMP-13 in cartilages and synovial tissues in CIA mice. Consistent with our *in vivo* findings, astragalin also profoundly inhibited TNFα-induced production of MMP-1, MMP-3, and MMP-13 in cultured MH7A cells. Taken together, these findings suggested that the joint-protective properties of astragalin may be mainly attributed to the down-regulation of MMPs expression.

Stimulation of RA-FLSs with TNF-α or IL-1 *in vitro* increases MMPs production via transcriptional activation (Burrage et al., 2006). AP-1 binding sites have been found in the promoter region of all MMPs and AP-1 appears to play a crucial role in the transcriptional activity of MMPs (Shiozawa et al., 1997; Han et al., 2001; Sweeney et al., 2005). c-Jun and c-Fos are two major components of AP-1 in the stimulated RA-FLSs and mediate the function of AP-1 (Han et al., 1998). Recent evidence suggests that interfering with AP-1 decoy oligonucleotides or using c-Fos/AP-1 inhibitors reduced the severity of CIA characterized by inflammatory cytokine overproduction and MMP synthesis (Aikawa et al., 2008), thus validating the crucial role of AP-1 in joint inflammation. Here, we demonstrated that astragalin significantly inhibited the activation of c-Jun, but not the c-Fos in TNF-α-induced MH7A cells. Therefore, we speculated that the joint-protective properties of astragalin are related to interfering with the AP-1 pathway.

AP-1 belongs to the class of basic leucine zipper transcription factors. Its activity can be markedly increased in synoviocytes as a response to the stimulation of cytokines, such as IL-1β and TNF-α, through MAPK, AKT, and other signaling pathways (Yamanishi and Firestein, 2001). Activation of p38 and JNK is commonly responsible for AP-1 activation through the phosphorylation of c-Jun. Next, we explored the effects of astragalin on the MAPK signaling pathway.

The MAPK pathway is central to many host responses and is one of the major signaling pathway-transmitting signals to immediate early genes implicated in the regulation of cytokine responses (Hammaker et al., 2003). Regulation of cytokine production in RA was directly associated with activation of MAPKs. Of the three MAPK families, JNK and p38 are highly active in RA-FLSs and also involved in the regulation of MMPs expression (Schett et al., 2000; Yan and Boyd, 2007; Yoshizawa et al., 2008; Guma and Firestein, 2012). Moreover, in RA-FLSs, TNF-α can bind to its surface receptors and activate the MAPK pathways, leading to the expression of multiple cytokines and chemokines, as well as the secretion of MMPs that contribute to tissue destruction (Li et al., 2017; Huang et al., 2018). As the upstream of AP-1, MAPK signaling may also be influenced by astragalin. Our results demonstrated that astragalin treatment effectively hindered the TNF-α-induced phosphorylation of MAPK in RA-FLSs, including p38 and JNK, but not ERK, in a dose-dependent manner. These findings suggested that the decreased activations of AP-1 and MAPK might account for the beneficial effects of astragalin in RA.

In summary, our study demonstrated that astragalin markedly ameliorated synovial inflammation and joint destruction in CIA mice. The joint-protective properties of astragalin could be mainly attributed to the down-regulation of MMPs expression by inhibiting the JNK/p38/AP-1 pathways. Taken together, astragalin may serve as an effective therapeutic drug for RA.

## Author Contributions

QL, QS, and YW conceived and designed the experiments. QJ and TW performed the research. HX analyzed the result. XW and YL wrote the manuscript draft. All authors have read and approved the submitted version.

## Conflict of Interest Statement

The authors declare that the research was conducted in the absence of any commercial or financial relationships that could be construed as a potential conflict of interest.
